# Vibration of symmetrically layered angle-ply cylindrical shells filled with fluid

**DOI:** 10.1371/journal.pone.0219089

**Published:** 2019-07-03

**Authors:** Nurul Izyan Mat Daud, K. K. Viswanathan

**Affiliations:** 1 UTM Centre for Industrial and Applied Mathematics, Ibnu Sina Institute for Scientific & Industrial Research, Universiti Teknologi Malaysia, Johor Bahru, Johor, Malaysia; 2 Department of Mathematical Sciences, Faculty of Science, Universiti Teknologi Malaysia, Johor Bahru, Johor, Malaysia; 3 Department of Mathematics, Kuwait College of Science and Technology, Doha District, Safat, Kuwait; University of New South Wales, AUSTRALIA

## Abstract

Vibrational behaviour of symmetric angle-ply layered circular cylindrical shell filled with quiescent fluid is presented. The equations of motion of cylindrical shell in terms of stress and moment resultants are derived from the first order shear deformation theory. Irrotational of inviscid fluid are expressed as the wave equation. These two equations are coupled. Strain-displacement relations and stress-strain relations are adopted into the equations of motion to obtain the differential equations with displacements and rotational functions. A system of ordinary differential equation is obtained in one variable by assuming the functions in separable form. Spline of order three is applied to approximate the displacement and rotational functions, together with boundary conditions, to get a generalised eigenvalue problem. The eigenvalue problem is solved for eigen frequency parameter and associate eigenvectors of spline coefficients. The study of frequency parameters are analysed using the parameters the thickness ratio, length ratio, angle-ply, properties of material and number of layers under different boundary conditions.

## Introduction

Composite materials are widely used in petroleum, chemical industry etc., due to the high stiffness and strength. Other characteristics of composite materials are resistance to corrosion and light in weight. The applications of these kinds of materials can be seen in industries like transportation, aircraft, construction, marine and consumer products. Hence, the investigation on the vibrational behaviour of the structure is carried out in order to determine its natural frequency. The important of finding frequency is to prevent the structure from resonance thus to improve the life-span of the structure. There are many literature studied the vibrational behaviour of the shell structure especially in cylindrical shell itself by considering different theories and method of solution. Warburton and Higgs [1] used Rayleigh-Ritz method to solve a cantilever cylindrical shell by considering Flugge’s shell theory. Using the same method, Song et al. [2] investigated the free vibration of symmetrically laminated composite cylindrical shell with arbitrary boundary conditions.

Among the researchers those who used Finite Element Method (FEM) in their analysis were Sivadas and Ganesan [3], Lam and Wu [4]. The wave propagation method was applied by Li [5], Zhang [6] and Iqbal et al. [7]. Lopatin and Morozov [8] solved the problem of a cantilever composite cylindrical shell using Galerkin method to find the fundamental frequencies. In addition, Haar wavelet method was used by Xie et al. [9] to analyse the free vibration of cylindrical shell based on Goldenveizer-Novozhilov shell theory. Meanwhile, Jin et al. [10] applied First Order Shear Deformation Theory (FSDT) to examine the vibration of functionally graded cylindrical shell. A spline strip method was adopted in studying the vibration of cross-ply laminated cylindrical shells [11]. Bickley-type spline method was applied to analyse the frequencies of free vibration of cylindrical shell [12–15]. Besides that, a spline-based differential quadrature method was used by Javidpoor et al. [16] and Ghasemi [17]. Ferreira et al. [18] used multiquadric radial basis function method to determine the natural frequencies of doubly curved cross-ply composite shells.

Moreover, the structure can also interact with fluid either the structure filled with, partially with, or submerged. The fluid can be flowing or non-flowing fluid. This type of investigation is known as fluid structure interaction. The characteristics of fluid itself affect the behaviour of the structure. Hence, the investigation has to consider both shell and fluid in order to get better results. Recently, the investigation on fluid structure interaction has get attention among the researchers.

Finite Element Method (FEM) was applied in the problem free vibration of isotropic, vertical cylindrical shell partially and completely filled with stationary liquid [19]. The shell equations were constructed using Sanders’ thin shell theory. Selmane and Lakis [20] applied FEM in solving the vibration of an anisotropic cylindrical shell submerged and subjected simultaneously to an internal and external flow by considering Sanders’ thin shell theory. Other literatures applied FEM in their analysis are Kochupillai et al. [21], Kochupillai et al. [22], Toorani and Lakis [23], Toorani and Lakis [24].

Gunawan et al. [25] conducted a study on cylindrical shells filled with fluid based on elastic foundation by considering Sanders’ thin shell theory. Krishna and Ganesan [26] investigated the results of free vibration of cylindrical shells filled with fluid. The shell was governed by first order deformation theory. Lakis et al. [27] analysed the isotropic and anisotropic plates and shells with and without fluid for linear and nonlinear vibration with the shell equations were based on Sanders’ shell theory and dynamic pressure of fluid was derived from Bernoulli’s equation. Galerkin method was used by Goncalves et al. [28] in solving the nonlinear dynamic behaviour of cylindrical shells filled with fluid with Donnell’s nonlinear shallow shell theory.

A study on the vibration of vertical circular cylindrical shell partially filled by an incompressible, compressible, quiescent and inviscid fluid using Rayleigh-Ritz method was analysed by Amabili [29]. The shell was constrained by simply-supported boundary conditions. In addition to that, Kwak et al. [30] studied the clamped-free cylindrical shell partially submerged in fluid using Sanders’ shell theory.

Vibrational analysis of fluid filled double-walled carbon nanotubes using the wave propagation method was carried out by Natsuki et al. [31] and the shell equations were based on simplified Flügge shell theory. Iqbal et al. [32] applied Love’s thin shell theory to study the vibration of a Functionally Graded Material (FGM) circular cylindrical shell filled with fluid and the fluid was an incompressible non-viscous fluid. A study on cylindrical shells filled with fluid resting on elastic foundations was carried out by Shah et al. [33] using wave propagation method. The shell was constrained with simply supported at the both ends and the Love’s thin shell theory was applied to the problem. A nonlinear vibration of cantilevered circular cylindrical containing quiescent fluid based on Flügge's shell theory and the fluid motion was modelled by linearized potential flow theory was investigated by Paak et al. [34].

A dynamic stiffness method was studied by Tran and Manh [35] to investigate the free vibration of cross-ply laminated composite circular cylindrical shells filled with fluid partially and also complete filling with fluid under clamped-free boundary conditions. Reissner-Mindlin theory was used for the shell equations. The fluid considered as non-viscous and incompressible. In addition, spline method was applied to solve the free vibration of layered cylindrical shell filled with fluid using Love’s thin shell theory [36]. Nurul Izyan et al. [37] applied the spline method in their analysis to determine the frequencies of anti-symmetric angle-ply laminated composite cylindrical shell filled with fluid. The shells’ equations were formulated based on FSDT.

According to a nonlocal theory, there are two types of models which are structural hardening and softening models. Li et al. [38] and Shen and Li [39] proved that both the hardening and softening models are correct and they are related to different types of surface effects, i.e. relaxation or tension of surface atomic lattices. Consequently, the two different models are caused by the long range attractive and repulsive interactions in pair potentials between atoms, respectively.

Different approach study is done in order to study the fluid structure interaction. The aim of this study is to determine the vibrational behaviour of symmetric angle-ply laminated composite cylindrical shell. The quiescent fluid-filled shell is considered. The quiescent fluid means that the vibration of fluid is depends on the vibration of the shell. Once the shell vibrates, the fluid will vibrate accordingly. Bickley-type spline is used to approximate the displacements and rotational functions since it gives better accuracy and also uses of lower order approximation in solving boundary value problem [40]. The shells’ equations are based on FSDT. Three and five layered shell under clamped-clamped and simply-supported boundary conditions are studied. The material properties of Kevler-49 Epoxy (KGE) and AS4/3501-6 Graphite/Epoxy (AGE) are used. Parametric studies on shell geometry i.e, length and thickness of the shells, type of materials, ply orientations, number of layer of the materials, and boundary conditions on frequencies are studied.

## Formulation of the problem

### Equations of shell

**Fig 1:**
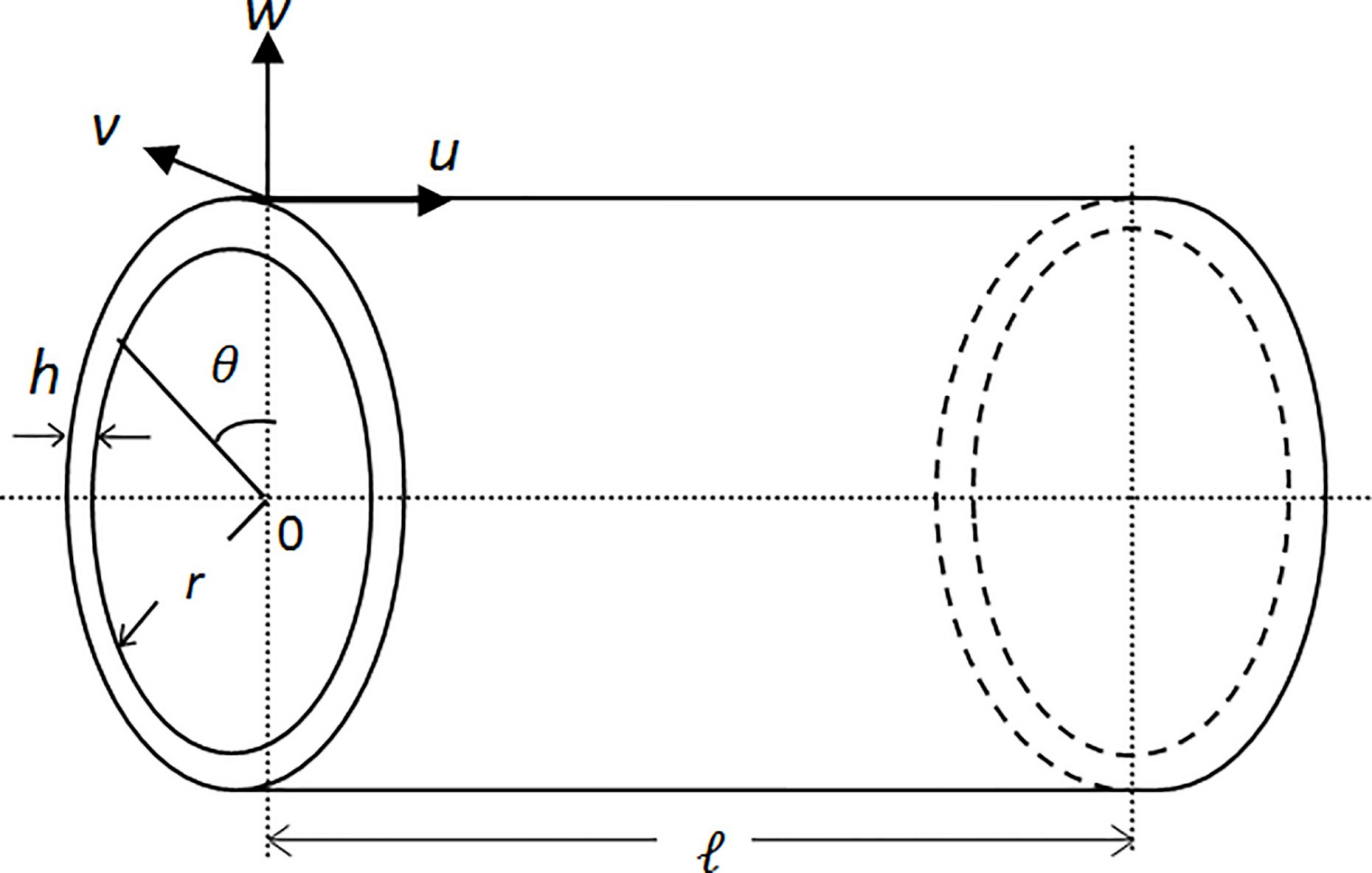
A geometry and coordinate system of a circular cylindrical shell.

Fig 1 shows the geometry of the circular cylindrical shell with the shell coordinates defined as (*x*,*θ*,*z*) where *x* is along the axial direction, *θ* is in the circumferential and *z* is along the normal direction. The length of the shell is l, thickness is *h* and radius is *r*. The displacement components *u*,*v*,*w* are expressed under the first order shear deformation theory [41] as

$$\begin{array}{l} u(x,\theta ,z,t)=u_{0}(x,\theta ,t)+z\psi_{x}(x,\theta ,t),\\ v(x,\theta ,z,t)=v_{0}(x,\theta ,t)+z\psi_{\theta }(x,\theta ,t),\\ w(x,\theta ,z,t)=w_{0}(x,\theta ,t), \end{array}$$ (1)

where *u*,*v*,*w* are the displacements in *x*,*θ*,*z* directions. *u*_0_,*v*_0_,*w*_0_ are mid-plane displacements and *ψ*_*x*_,*ψ*_*θ*_ are the rotational functions of the normal to the mid-plane with respect to the *x*- and *θ*- axes. Equations of motion for cylindrical shell are formulated using FSDT [42, 43]. The equations of cylindrical shell which included fluid are written as

$$\begin{array}{l} \frac{\partial N_{x}}{\partial x}+\frac{1}{r}\frac{\partial N_{\theta x}}{\partial \theta }=I_{1}\frac{\partial^{2}u}{\partial t^{2}},\\ \frac{\partial N_{x\theta }}{\partial x}+\frac{1}{r}\frac{\partial N_{\theta }}{\partial \theta }+\frac{1}{r}Q_{\theta }=I_{1}\frac{\partial^{2}v}{\partial t^{2}},\\ \frac{\partial Q_{x}}{\partial x}+\frac{1}{r}\frac{\partial Q_{\theta }}{\partial \theta }-\frac{1}{r}N_{\theta }=I_{1}\frac{\partial^{2}w}{\partial t^{2}}-p,\\ \frac{\partial M_{x}}{\partial x}+\frac{1}{r}\frac{\partial M_{x\theta }}{\partial \theta }-Q_{x}=I_{3}\frac{\partial^{2}\psi_{x}}{\partial t^{2}},\\ \frac{\partial M_{x\theta }}{\partial x}+\frac{1}{r}\frac{\partial M_{\theta }}{\partial \theta }-Q_{\theta }=I_{3}\frac{\partial^{2}\psi_{\theta }}{\partial t^{2}}. \end{array}$$ (2)

Here, *p* is the fluid pressure. *I*_1_ and *I*_3_ are the normal and rotary inertia coefficients, given by [42,43].

$$(I_{1},I_{3})=\int \rho^{\left(k\right)}(1,z^{2})dz,$$ (3)

where *ρ*^(*k*)^ is the material density of the *k*-th layer of the shell.

Stress and moment resultants are expressed as

$$\begin{array}{l} (N_{x},N_{\theta },N_{x\theta },Q_{x},Q_{\theta })=\int_{z}\left(\sigma_{x},\sigma_{\theta },\tau_{x\theta },\tau_{xz},\tau_{\theta z}\right)dz,\\ (M_{x},M_{\theta },M_{x\theta })=\int_{z}\left(\sigma_{x},\sigma_{\theta },\tau_{x\theta }\right)zdz. \end{array}$$ (4)

Using stress-strain relations and strain-displacement relations of the *k*-th layer by neglecting the transverse normal strain and stress, the stress and moment resultants are obtained as follows

$$\left(\begin{array}{c} N_{x}\\ N_{\theta }\\ N_{x\theta }\\ M_{x}\\ M_{\theta }\\ M_{x\theta } \end{array}\right)=\left(\begin{array}{cccccc} A_{11} & A_{12} & A_{16} & B_{11} & B_{12} & B_{16}\\ A_{12} & A_{22} & A_{26} & B_{12} & B_{22} & B_{26}\\ A_{16} & A_{26} & A_{66} & B_{16} & B_{26} & B_{66}\\ B_{11} & B_{12} & B_{16} & D_{11} & D_{12} & D_{16}\\ B_{12} & B_{22} & B_{26} & D_{12} & D_{22} & D_{26}\\ B_{16} & B_{26} & B_{66} & D_{16} & D_{26} & D_{66} \end{array}\right)\left(\begin{array}{l} \frac{\partial u_{0}}{\partial x}\\ \frac{1}{r}\frac{\partial v_{0}}{\partial \theta }+\frac{w}{r}\\ \frac{1}{r}\frac{\partial u_{0}}{\partial \theta }+\frac{\partial v}{\partial x}\\ \frac{\partial \psi_{x}}{\partial x}\\ \frac{1}{r}\frac{\partial \psi_{\theta }}{\partial \theta }\\ \frac{1}{r}\frac{\partial \psi_{x}}{\partial \theta }+\frac{\partial \psi_{\theta }}{\partial x} \end{array}\right),$$ (5)

and

$$\left(\begin{array}{l} Q_{\theta }\\ Q_{x} \end{array}\right)=K\left(\begin{array}{cc} A_{44} & A_{45}\\ A_{45} & A_{55} \end{array}\right)\left(\begin{array}{l} \psi_{\theta }+\frac{1}{r}\frac{\partial w}{\partial \theta }-\frac{v_{0}}{r}\\ \psi_{x}+\frac{\partial w}{\partial x} \end{array}\right).$$ (6)

Here *A*_*ij*_, *B*_*ij*_ and *D*_*ij*_ are extensional rigidities, bending-stretching coupling rigidities and bending rigidities, respectively and defined as

$$\begin{array}{l} A_{ij}=\sum_{k=1}^{N-1}Q_{ij}^{\left(k\right)}\left(z_{k}-z_{k-1}\right),\;B_{ij}=\frac{1}{2}\sum_{k=1}^{N-1}Q_{ij}^{\left(k\right)}\left(z^{2}{}_{k}-z^{2}{}_{k-1}\right),\\ D_{ij}=\frac{1}{3}\sum_{k=1}^{N-1}Q_{ij}^{\left(k\right)}\left(z^{3}{}_{k}-z^{3}{}_{k-1}\right),\;(i,j=1,2,6), \end{array}$$

and

$$A_{ij}=K\sum_{k=1}^{N-1}Q_{ij}{}^{\left(k\right)}(z_{k}-z_{k-1}),\;(i,j=4,5).$$

Here *z*_*k*_ and *z*_*k*−1_ are boundaries of the *k*-th layer and *K* is the shear correction factor which is depends on lamination scheme [44, 45]. Since shell is considered to be a symmetric angle-ply, therefore, the coefficients *A*_16_,*A*_26_,*A*_45_,*D*_16_,*D*_26_ and *B*_*ij*_ are identically zeroes [13].

The displacements *u*_0_, *v*_0_, *w* and shear rotations *ψ*_*x*_,*ψ*_*θ*_ are assumed in the form of

$$\begin{array}{l} u_{0}(x,\theta ,t)=U(x)\cos n\theta e^{i\omega t},\\ v_{0}(x,\theta ,t)=V(x)\sin n\theta e^{i\omega t},\\ w(x,\theta ,t)=W(x)\cos n\theta e^{i\omega t},\\ \psi_{x}(x,\theta ,t)=\Psi_{x}(x)\cos n\theta e^{i\omega t},\\ \psi_{\theta }(x,\theta ,t)=\Psi_{\theta }(x)\sin n\theta e^{i\omega t}, \end{array}$$ (7)

in which *ω*,*t* and *n* are the angular frequency, time and circumferential node number respectively.

Non-dimensional parameters used are as follows

$$\begin{array}{l} L=\frac{l}{r};a\;\mathrm{length}\;\mathrm{parameter,}\\ X=\frac{x}{l};a\;\mathrm{distance}\;\mathrm{coordinate},\\ R=\frac{r}{l};a\;\mathrm{radius}\;\mathrm{parameter},\\ H=\frac{h}{r};\mathrm{the}\;\mathrm{thickness}\;\mathrm{parameter},\\ \lambda =\omega l\sqrt{\frac{I_{1}}{A_{11}}};a\;\mathrm{frequency}\;\mathrm{parameter},\\ \delta_{k}=\frac{h_{k}}{h};a\;\mathrm{relative}\;\mathrm{layer}\;\mathrm{thickness}\;\mathrm{of}k‐\mathrm{th}\;\mathrm{layer}. \end{array}$$ (8)

Substituting Eqs (5) and (6) into Eq (2), then, using Eqs (7) and (8), the differential equations are obtained in terms of displacement and rotational functions and it can be written in the matrix form as follows

$$\left(\begin{array}{ccccc} L_{11} & L_{12} & L_{13} & L_{14} & L_{15}\\ L_{22} & L_{22} & L_{23} & L_{24} & L_{25}\\ L_{31} & L_{32} & L_{33} & L_{34} & L_{35}\\ L_{41} & L_{42} & L_{43} & L_{44} & L_{45}\\ L_{51} & L_{52} & L_{53} & L_{54} & L_{55} \end{array}\right)\left(\begin{array}{l} U\\ V\\ W\\ \Psi_{X}\\ \Psi_{\theta } \end{array}\right)=\left(\begin{array}{l} 0\\ 0\\ 0\\ 0\\ 0 \end{array}\right),$$ (9)

where *L*_*ij*_ are differential operators given as follows

$$\begin{array}{l} L_{11}=\frac{d^{2}}{dX^{2}}-S_{10}\frac{n^{2}}{R^{2}}+\lambda^{2},L_{12}^{}=\left(S_{2}+S_{10}\right)\frac{n}{R}\frac{d}{dX},L_{13}^{}=S_{2}\frac{1}{R}\frac{d}{dX},\\ L_{14}^{}=L_{15}^{}=0,L_{21}^{}=-\left(S_{2}+S_{10}\right)\frac{n}{R}\frac{d}{dX},L_{22}^{}=S_{10}\frac{d^{2}}{dX^{2}}-\left(S_{3}\frac{n^{2}}{R^{2}}+KS_{13}\frac{1}{R^{2}}\right)+\lambda^{2},\\ L_{23}^{}=-\left(S_{3}+KS_{13}\right)\frac{n}{R^{2}}=L_{32}^{},L_{24}^{}=0,L_{25}^{}=KS_{13}\frac{1}{R},L_{31}^{}=-S_{2}\frac{1}{R}\frac{d}{dX},\\ L_{33}^{}=KS_{14}\frac{d^{2}}{dX^{2}}-\left(S_{3}\frac{1}{R^{2}}+KS_{13}\frac{n^{2}}{R^{2}}\right)+\left(1+\frac{\rho_{f}}{I_{1}}\frac{J_{n}(R)}{J_{n}^{\prime }(R)}\right)\lambda^{2},\\ L_{34}^{}=KS_{14}\frac{d}{dX},L_{35}^{}=KS_{13}\frac{n}{R},L_{41}^{}=L_{42}^{}=0,L_{43}^{}=-KS_{14}\frac{d}{dX},\\ L_{44}^{}=S_{7}\frac{d^{2}}{dX^{2}}-\left(S_{12}\frac{n^{2}}{R^{2}}+KS_{14}\right)+\lambda^{2}p_{1},L_{45}^{}=\left(S_{12}+S_{8}\right)\frac{n}{R}\frac{d}{dX},\\ L_{51}^{}=0,L_{52}^{}=KS_{13}\frac{1}{R},L_{53}^{}=KS_{13}\frac{n}{R},L_{54}=-\left(S_{12}+S_{8}\right)\frac{n}{R}\frac{d}{dX},\\ L_{55}^{}=S_{12}\frac{d^{2}}{dX^{2}}-\left(S_{9}\frac{n^{2}}{R^{2}}+KS_{13}\right)+\lambda^{2}p_{1}, \end{array}$$

with

$$\begin{array}{l} S_{2}=\frac{A_{12}}{A_{11}},S_{3}=\frac{A_{22}}{A_{11}},S_{7}=\frac{D_{11}}{l^{2}A_{11}},S_{8}=\frac{D_{12}}{l^{2}A_{11}},S_{9}=\frac{D_{22}}{l^{2}A_{11}},S_{10}=\frac{A_{66}}{A_{11}},\\ S_{12}=\frac{D_{66}}{l^{2}A_{11}},S_{13}=\frac{A_{44}}{A_{11}},S_{14}=\frac{A_{55}}{A_{11}},P_{1}=\frac{I_{3}}{l^{2}I_{1}}. \end{array}$$

### Equation of fluid

The fluid pressure, *p* is obtained as an explicit expression and coupled to the equations of the shell through radial displacement. The wave equation is obtained for irrotational flow of an inviscid fluid undergoing small oscillations in the cylindrical coordinates system (*x*, *θ*, *r*) [46] as

$$\frac{\partial^{2}p}{\partial r^{2}}+\frac{1}{r}\frac{\partial p}{\partial r}+\frac{1}{r^{2}}\frac{\partial^{2}p}{\partial \theta^{2}}+\frac{\partial^{2}p}{\partial x^{2}}=\frac{\partial^{2}p}{c^{2}\partial t^{2}},$$ (10)

where *c* is the speed sound of the fluid.

The pressure is assumed in the separable form as

$$p(x,\theta ,r,t)=\psi (x)\cos(n\theta )J_{n}(r)e^{i\omega t}.$$ (11)

Here *J*_*n*_ is the Bessel function of order *n*.

The permeability condition on the fluid- shell interface ensures that the fluid remains in contact with the shell wall and written as

$${-\frac{1}{i\omega \rho_{f}}\frac{\partial p}{\partial r}|}_{r=R}={\frac{\partial w}{\partial t}|}_{r=R},$$ (12)

where *ρ*_*f*_ is the density of the fluid. Eq (12) is solved by using the displacement component in the normal direction given in the Eq (7) together with the Eq (11), hence, the following relation is obtained

$$\psi (x)=\frac{\omega^{2}\rho_{f}}{J_{n}^{\prime }(r)}W(x).$$ (13)

Then, by applying Eq (13) into Eq (11), the fluid pressure, *p* is obtained as follows

$$p(x,\theta ,r,t)=-\rho_{f}\frac{J_{n}(r)}{J_{n}^{\prime }(r)}\frac{\partial^{2}w}{\partial t^{2}}.$$ (14)

## Solution procedure

### Spline collocation method

The displacement functions *U*,*V*,*W* and rotational functions Ψ_*X*_ and Ψ_*θ*_ are approximated using cubic splines as

$$U^{*}\left(X\right)=\sum_{i=0}^{2}a_{i}X^{i}+\sum_{j=0}^{N-1}b_{j}{\left(X-X_{j}\right)}^{3}H\left(X-X_{j}\right),$$

$$V^{*}\left(X\right)=\sum_{i=0}^{2}c_{i}X^{i}+\sum_{j=0}^{N-1}d_{j}{\left(X-X_{j}\right)}^{3}H\left(X-X_{j}\right),$$

$$W^{*}\left(X\right)=\sum_{i=0}^{2}e_{i}X^{i}+\sum_{j=0}^{N-1}f_{j}{\left(X-X_{j}\right)}^{3}H\left(X-X_{j}\right).$$

$${\Psi_{X}}^{*}\left(X\right)=\sum_{i=0}^{2}g_{i}X^{i}+\sum_{j=0}^{N-1}p_{j}{\left(X-X_{j}\right)}^{3}H\left(X-X_{j}\right),$$

$${\Psi_{\theta }}^{*}\left(X\right)=\sum_{i=0}^{2}l_{i}X^{i}+\sum_{j=0}^{N-1}q_{j}{\left(X-X_{j}\right)}^{3}H\left(X-X_{j}\right).$$ (15)

Here, *H*(*X*−*X*_*j*_) is the Heaviside step function and *N* is the number of intervals in the range of *X*∈[0,1] is divided. These splines are lower order approximation and an effective method since it has fast convergence and high accuracy. The points of division $X=X_{s}=\frac{s}{N},(s=0,1,2,\ldots N)$ are chosen as the knots of the splines as well as the collocation points. Assuming that the differential equations given by Eq (9) are satisfied by these splines given in Eq (15) at the knots, a set of 5*N*+5 homogeneous equations into 5*N*+15 unknown spline coefficients *a*_*i*_,*b*_*j*_,*c*_*i*_,*d*_*j*_,*e*_*i*_,*f*_*j*_,*g*_*i*_,*p*_*j*_,*l*_*i*_,*q*_*j*_ (*i* = 0,1,2;*j* = 0,1,2,…,*N*−1) is obtained.

### Boundary conditions

Two types of boundary conditions are used to analyse the problem

- Clamped-Clamped (C-C)(both the ends are clamped);

$$U=0,V=0,W=0,\Psi_{X}=0,\Psi_{\theta }=0\;\mathrm{at}\;X=0\;\mathrm{and}\;X=1.$$

- Simply supported–Simply supported (S–S)(both the ends are simply supported);

$$V=0,W=0,\Psi_{\theta }=0,N_{x}=0,M_{x}=0\;\mathrm{at}\;X=0\;\mathrm{and}\;X=1.$$

The system will get ten more equations on spline coefficients by imposing any one of the boundary conditions. Combining these ten equations with the earlier 5*N*+5 homogeneous equations, one can get 5*N*+15 equations in the same number unknown coefficients. This can be written as

$$\left[P\right]\left\{q\right\}=\lambda^{2}\left[Q\right]\left\{q\right\}.$$ (16)

Here, [*P*] and [*Q*] are the square matrices of order (5*N*+15)×(5*N*+15). {q} is the column matrix of the spline coefficients of order (5*N*+15)×1 and λ is the frequency parameter.

## Results and discussion

### Convergence and comparative studies

The convergence study has been made in order to determine the number of iteration. This is done by fixing the circumferential node number, thickness ratio, length ratio, number of layers and orientation of the material under C-C and S-S boundary conditions. The number of knots, *N* is taken as 16 since the change in percentage for next following values of *N* is 0.29%.

**Fig 2:**
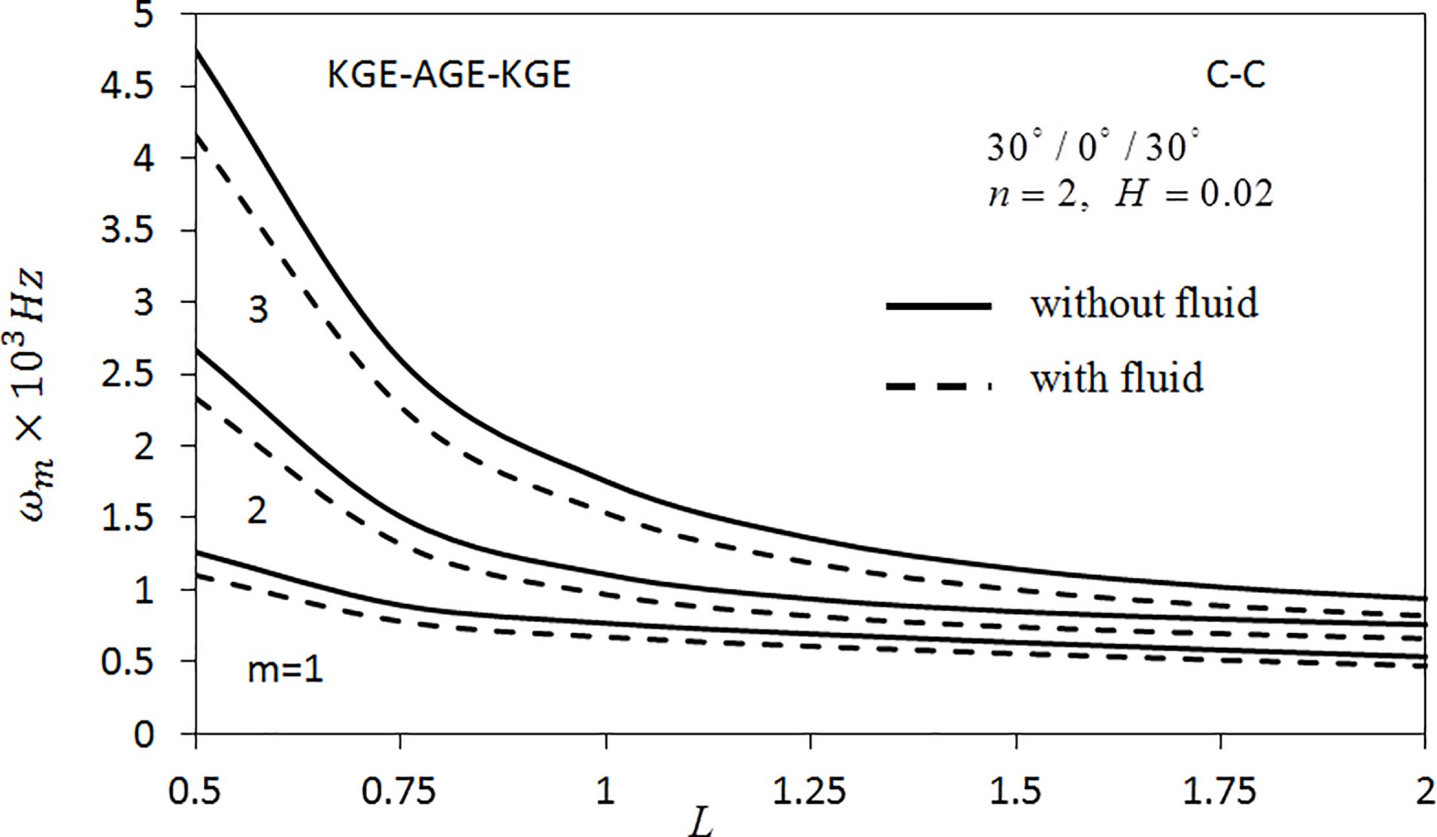
Effect of length parameter on the angular frequency of three layered symmetric angle-ply shells under C-C boundary conditions. Layer materials: KGE-AGE-KGE.

In the case of comparison studies, none of literature has been done on symmetric angle-ply shell with consideration of fluid. There are studies on symmetric angle-ply shells but limited to empty shells only [4, 13]. Hence, an investigation on the empty shell as well as the fluid-filled shells with respect to the length parameter is carried out as shown in Fig 2. The effects of the fluid on angular frequency *ω*_*m*_(*m* = 1,2,3) of three layered symmetric angle-ply shells using the materials Kevler-49 epoxy (KGE) and AS4/3501-6 Graphite/epoxy (AGE) are arranged in the order of KGE-AGE-KGE with the angle orientations at (30°/0°/30°) are presented. The parameters *n* = 2, *H* = 0.02 are fixed. The shell is clamped at both the ends. According to Fig 2, the frequency of fluid-filled shell is lower than the frequency of the empty shell. This result is expected as the fluid in the shell gives an added mass to the shell.

### Analysis

Three and five layered symmetric angle-ply circular cylindrical shells with fluid are analysed. The materials which are KGE and AGE are used [47]. The shear correction factor *K* = 5/6 is fixed throughout this analysis [44] and first three frequency parameters are studied for all the cases.

**Fig 3:**
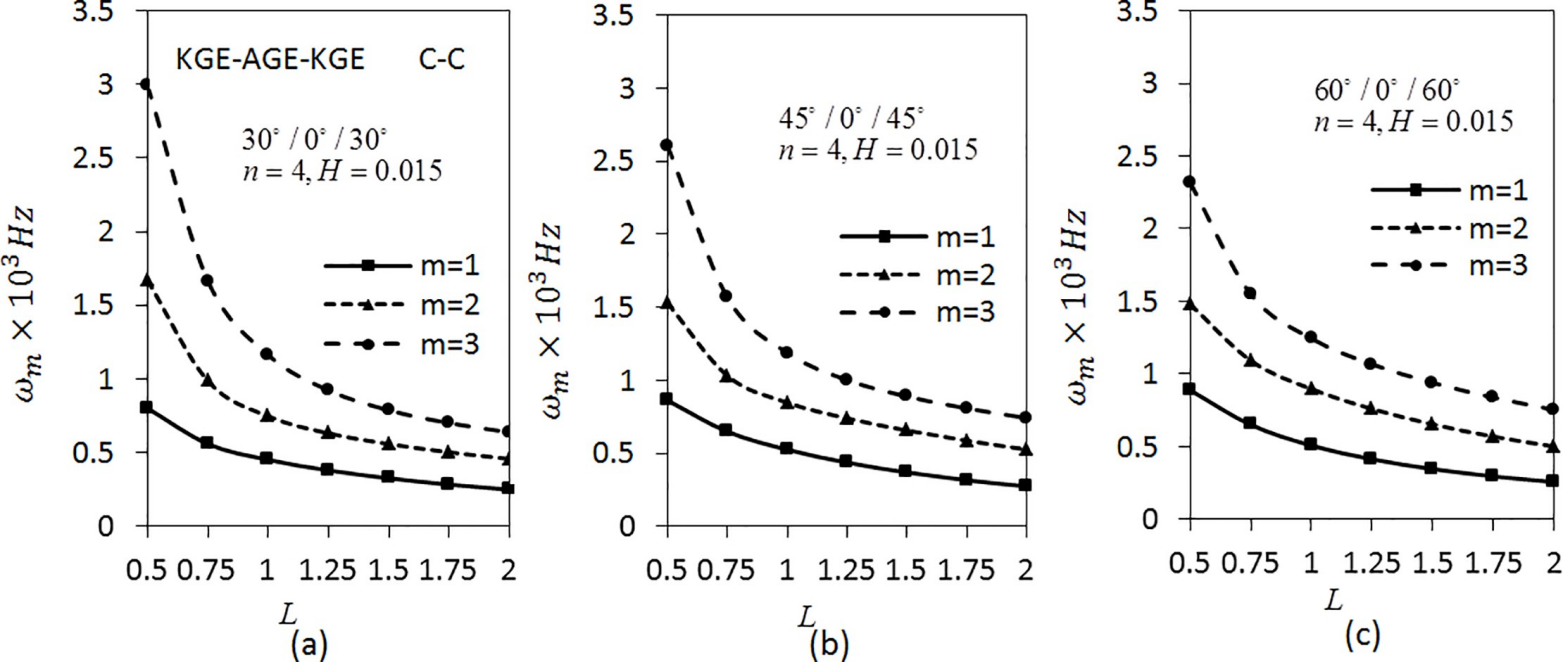
Effect of length parameter on the angular frequency of three layered symmetric angle-ply shells under C-C boundary conditions. Layer materials: KGE-AGE-KGE.

Fig 3 shows the variation of angular frequencies *ω*_*m*_(*m* = 1,2,3) on length parameter is studied for three layered shell with the combination of the materials KGE and AGE with C-C boundary conditions. The parameters *n* = 4 and *H* = 0.015 are fixed and the ply-angles are arranged as 30°/0°/30°, 45°/0°/45° and 60°/0°/60° using the materials AGE and KGE and ordered as KGE-AGE-KGE. Since the frequency parameter *λ*_*m*_ is explicitly a function of length of the cylinder, hence when studying the influence of the length of the cylinder on its vibrational behaviour, the angular frequency *ω*_*m*_(*m* = 1,2,3) is considered instead of *λ*_*m*_. The relationship between the frequency and the length of the shell can be seen as in [48]. The natural frequency or equivalent rigidity increases with increasing the non-dimensional scale parameter [49].

**Fig 4:**
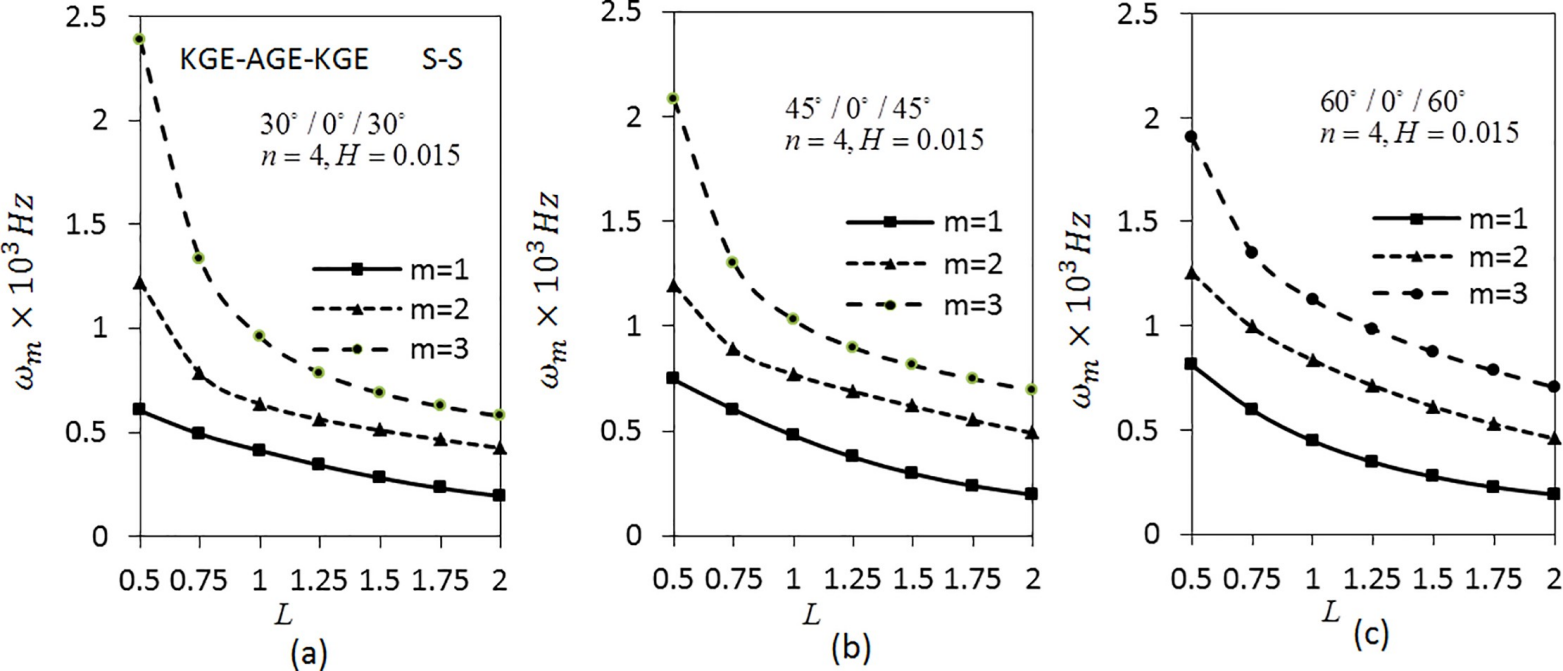
Effect of length parameter on the angular frequency of three layered symmetric angle-ply shells under S-S boundary conditions. Layer materials: KGE-AGE-KGE.

From the analysis, it can be seen that the frequency decreases as the length parameter increases and the frequency is higher for higher modes. The frequency decreases fast at *L* ranging from 0.5 to 0.75. Later, it decreases slowly. It also can be seen that the frequency for *m* = 1 of Fig 3(A) is the lowest compared to Fig 3(B) and 3(C). As for *m* = 2,3, the values of the frequencies are the lowest for Fig 3(C), followed by Fig 3(B) and 3(A). Further, investigation on S-S boundary conditions is presented in Fig 4 by fixing the parameters as in Fig 3. The trend shown by the graph of Fig 4 is similar to Fig 3. The results show that the frequencies obtained by S-S boundary conditions are lower than C-C boundary conditions.

**Fig 5:**
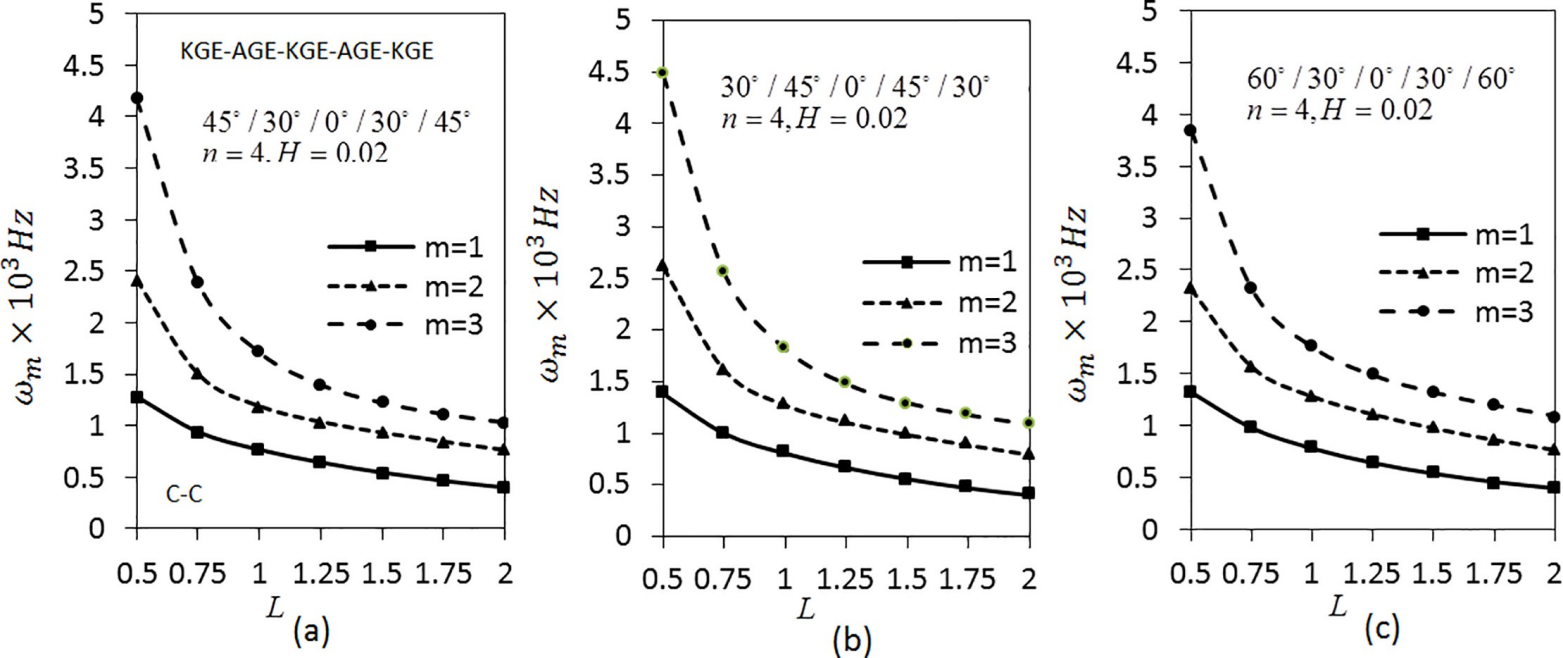
Effect of the length parameter on the angular frequency of five layered symmetric angle-ply shells under C-C boundary conditions. Layer materials: KGE-AGE-KGE-AGE-KGE.

**Fig 6:**
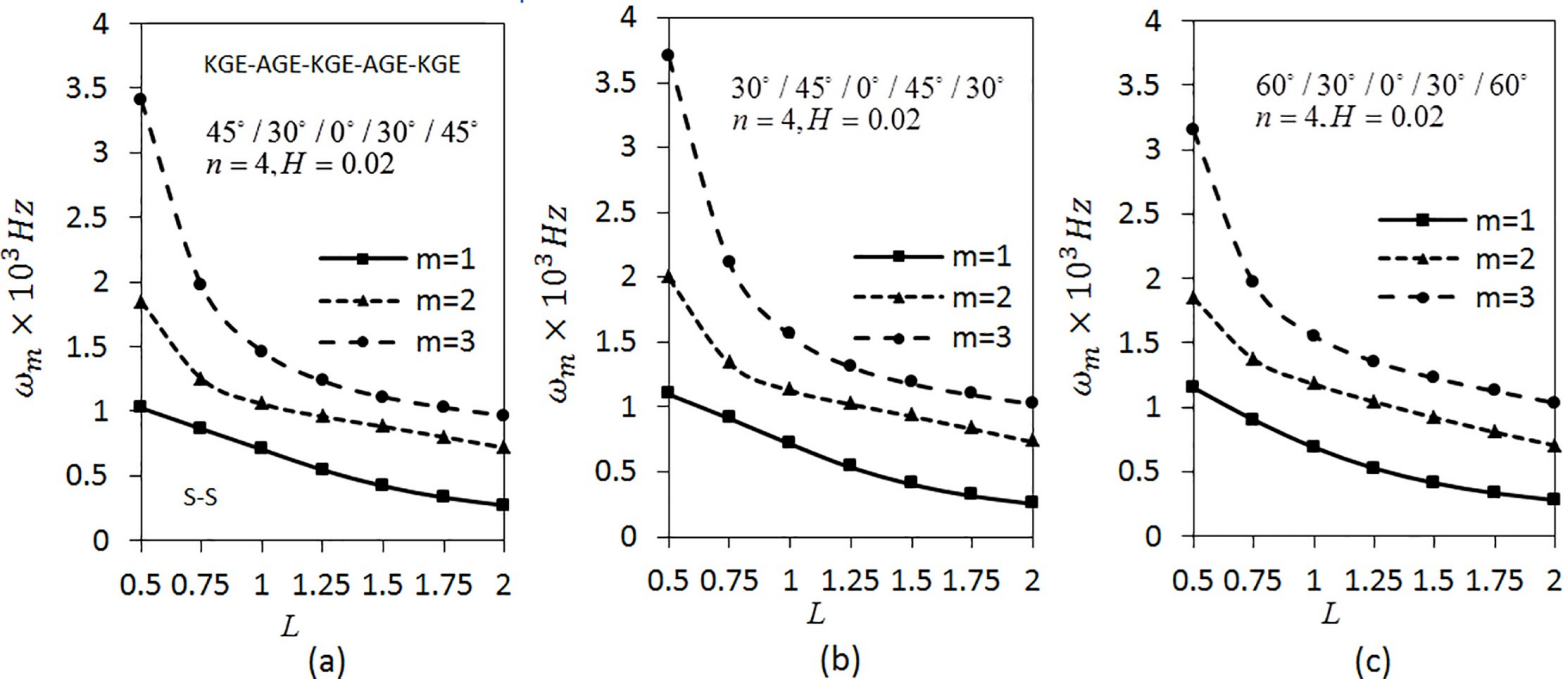
Effect of the length parameter on the angular frequency of five layered symmetric angle-ply shells under S-S boundary conditions. Layer materials: KGE-AGE-KGE-AGE-KGE.

Fig 5 corresponds to the effect of length parameter on angular frequency *ω*_*m*_(*m* = 1,2,3) of five layered angle-ply arranged materials in the order KGE-AGE-KGE-AGE-KGE with ply-angles of 45°/30°/0°/30°/45°, 30°/45°/0°/45°/30° and 60°/30°/0°/30°/60°, respectively for C-C conditions. The values of *n* = 4 and *H* = 0.02 is fixed. The figure shows that the frequency decreases as the length parameter increases. The investigation is continued by applying S-S boundary conditions using the same parameters as shown in Fig 6. The trend of graph is similar to the Fig 5. The values of *ω*_*m*_(*m* = 1,2,3) in Fig 6 are lower than the value of *ω*_*m*_(*m* = 1,2,3) in Fig 5.

**Fig 7:**
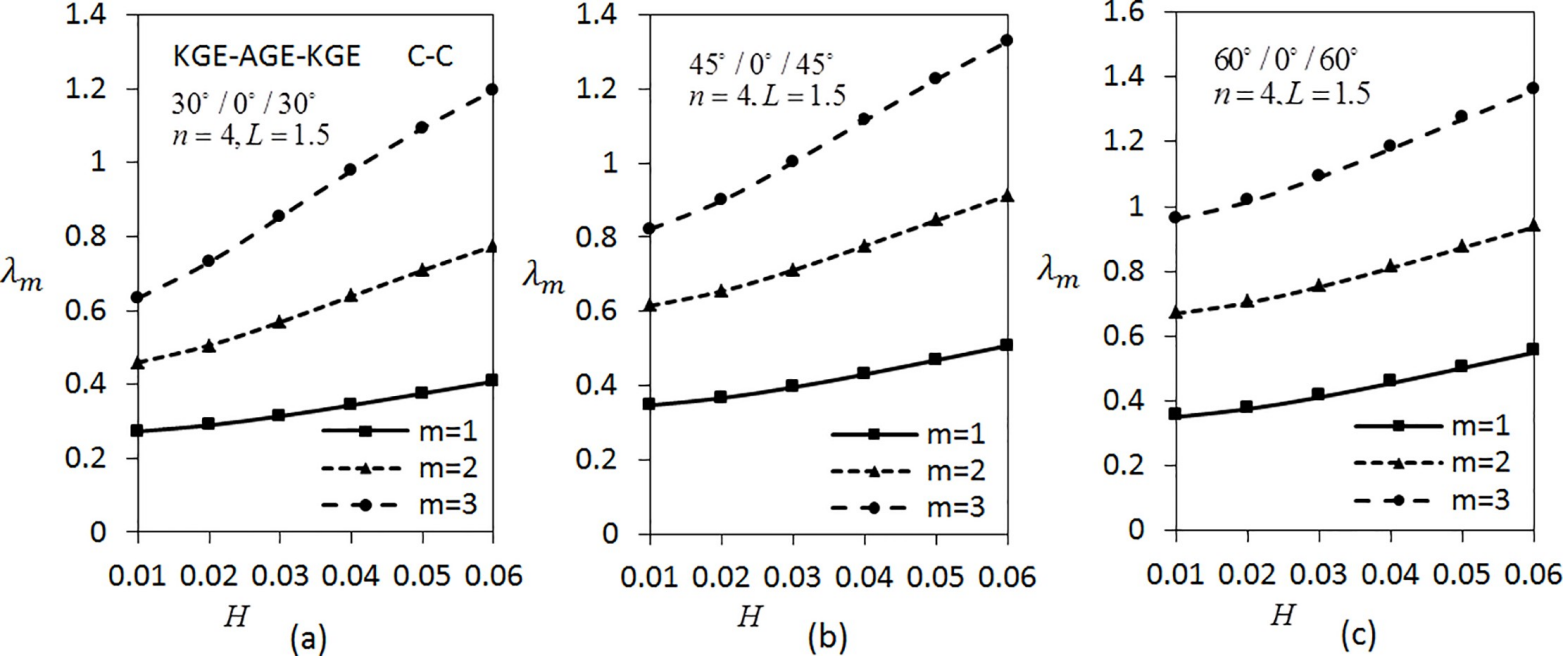
Effect of thickness parameter on the frequency for three layered symmetric angle-ply shells under C-C boundary conditions. Layer materials: KGE-AGE-KGE.

**Fig 8:**
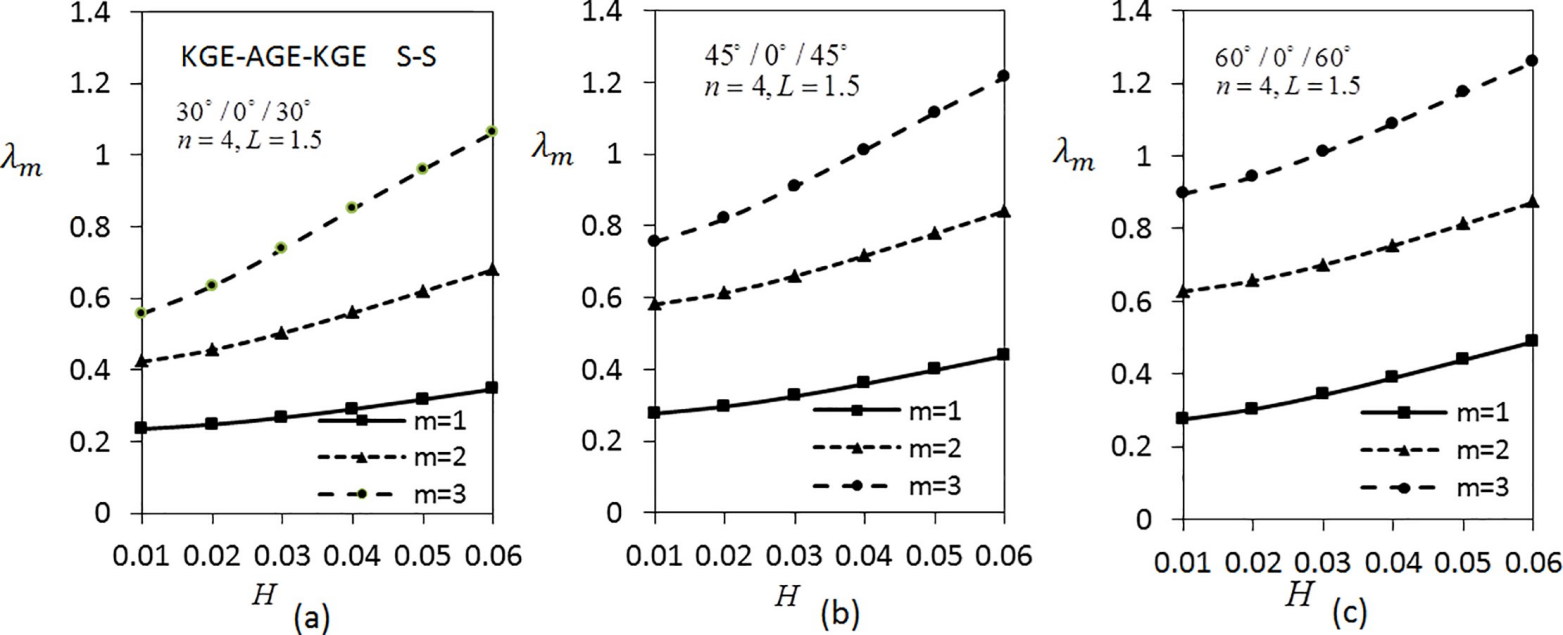
Effect of thickness parameter on frequency for three layered symmetric angle-ply shells under S-S boundary conditions. Layer materials: KGE-AGE-KGE.

Fig 7 shows the effect of thickness parameter on frequencies *λ*_*m*_(*m* = 1,2,3) by considering three layered shells arranged in KGE-AGE-KGE materials with *n* = 4 and *L* = 1.5 are fixed under C-C boundary conditions. The shell is arranged in the order of 30°/0°/30°, 45°/0°/45° and 60°/0°/60°. From the analysis, it can be seen clearly that the frequency rises as the thickness parameter rises. The frequencies are greater for larger modes. The values of *λ*_*m*_(*m* = 1,2,3) in Fig 7(A) are the lowest compared to Fig 7(B) and 7(C). In addition, Fig 8 presents the variation of frequencies *λ*_*m*_(*m* = 1,2,3) under S-S boundary conditions. Results show that the values of *λ*_*m*_(*m* = 1,2,3) in Fig 8 are lower than the values of *λ*_*m*_(*m* = 1,2,3) in Fig 7.

**Fig 9:**
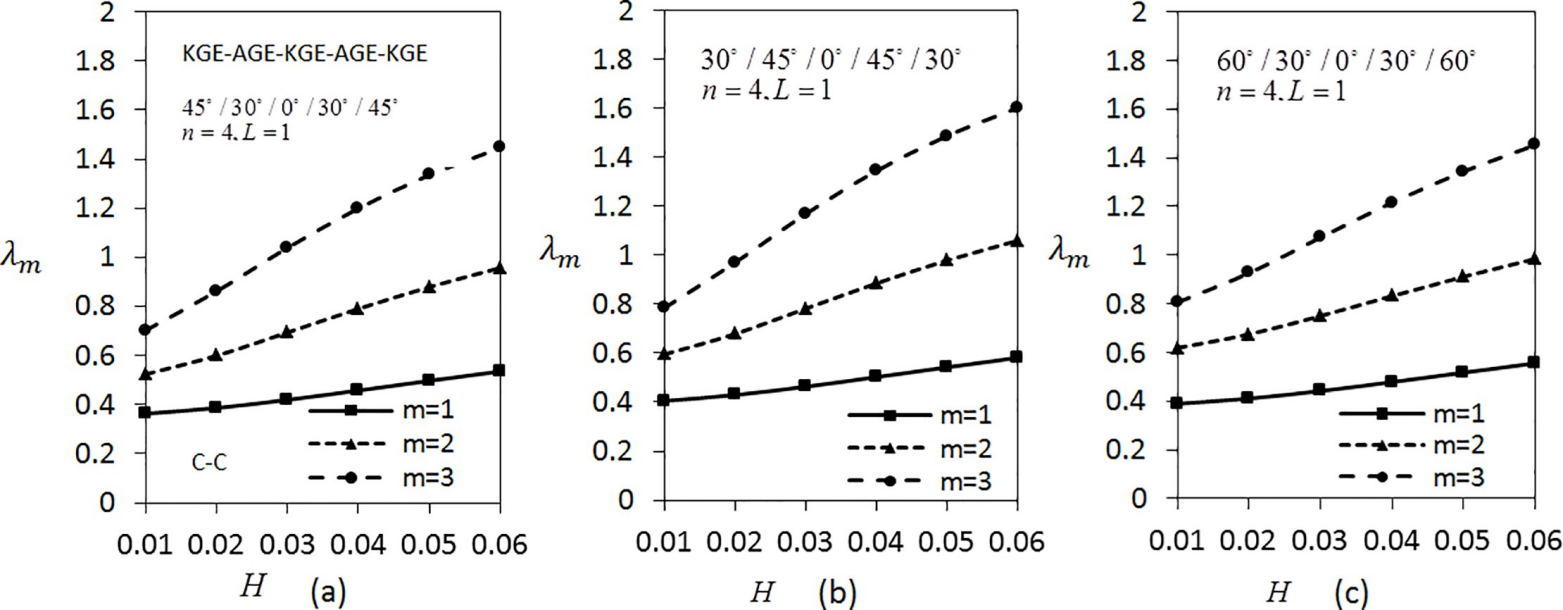
Effect of thickness parameter on frequency for five layered symmetric angle-ply shells under C-C boundary conditions. Layer materials: KGE-AGE-KGE-AGE-KGE.

**Fig 10:**
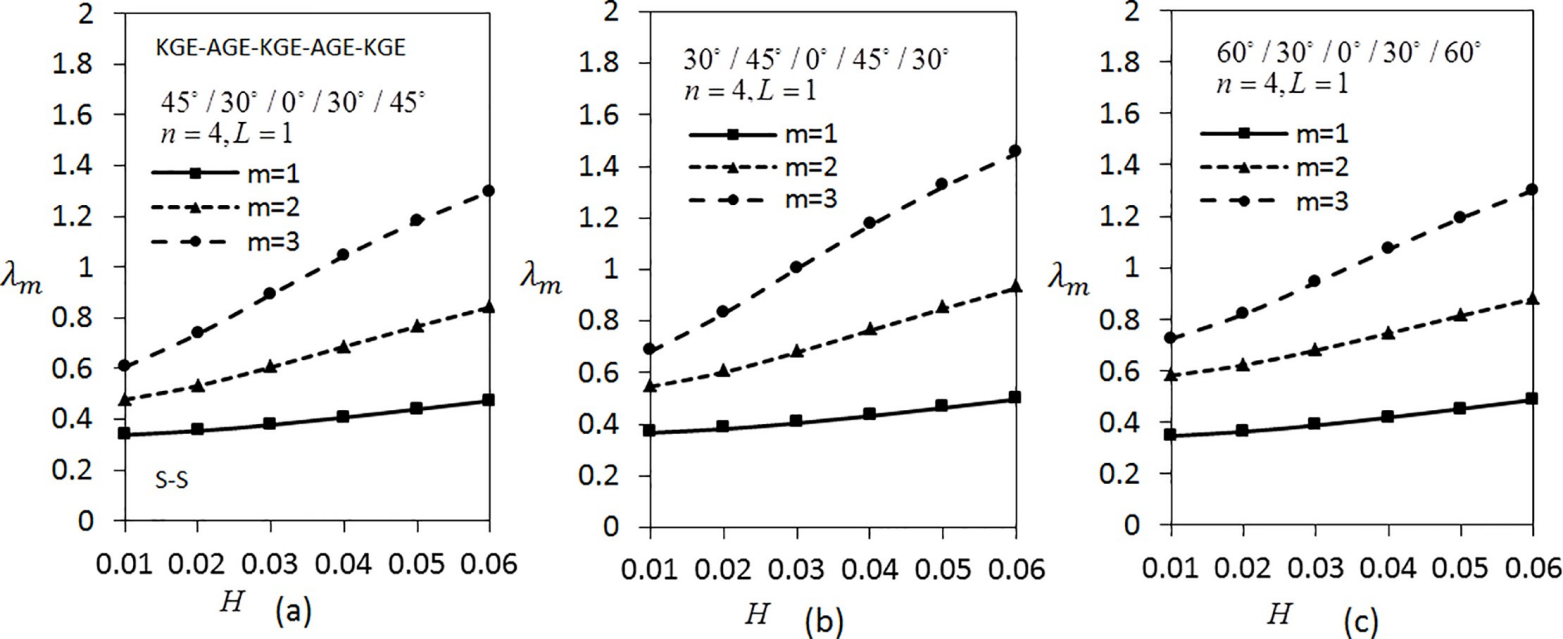
Effect of thickness parameter on frequency for five layered symmetric angle-ply shells under S-S boundary conditions. Layer materials: KGE-AGE-KGE-AGE-KGE.

Fig 9 shows the variation of *λ*_*m*_(*m* = 1,2,3) with respect to the thickness parameter for five layered angle-ply by fixing *n* = 4 and *L* = 1 for C-C boundary conditions. The materials are arranged as KGE-AGE-KGE-AGE-KGE with angle orientation at 45°/30°/0°/30°/45°, 30°/45°/0°/45°/30° and 60°/30°/0°/30°/60°. Clearly, the frequency is higher for higher values of thickness parameter. Fig 9(A) has the lowest frequency compared to Fig 9(B) and 9(C). Applying the same parameters as in Fig 9, investigation on S-S boundary conditions is carried out as depicted in Fig 10. It can be seen that Fig 10(A) has the lowest frequency, followed by Fig 10(B) and 10(C). It can be observed that the values of *λ*_*m*_(*m* = 1,2,3) under S-S boundary conditions are lower compared to the values of *λ*_*m*_(*m* = 1,2,3) under C-C boundary conditions.

**Fig 11:**
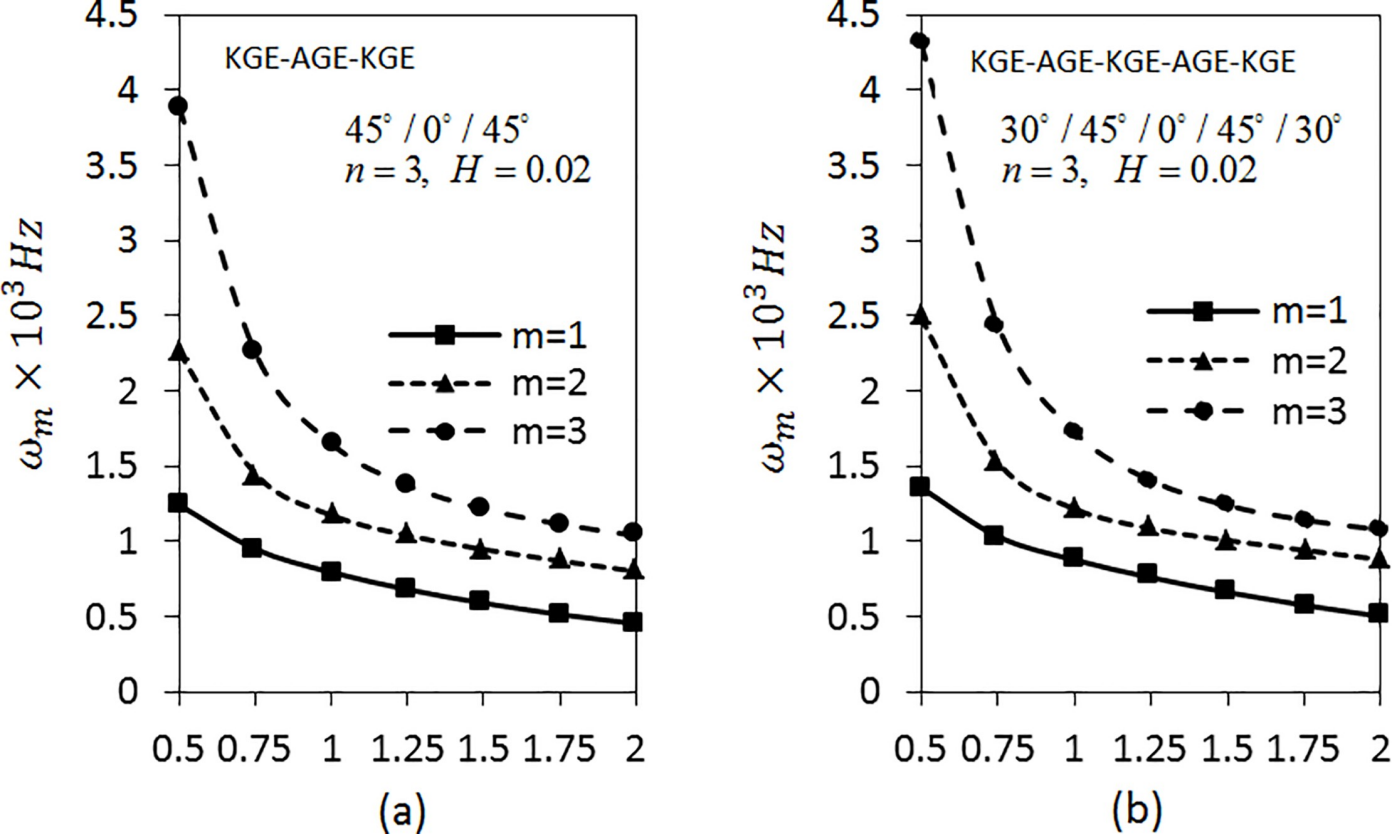
Effect of length parameter on frequency for three and five layered symmetric angle-ply shells under C-C boundary conditions.

By fixing *n* = 3, the effects of length parameter for both three and five layered shells on frequencies under C-C boundary conditions are investigated as depicted in Fig 11. The materials are arranged as KGE-AGE-KGE with angle orientation at 45°/0°/45° as shown in Fig 11(A). Meanwhile, Fig 11(B) used KGE-AGE-KGE-AGE-KGE materials with angle orientation at 30°/45°/0°/45/30°. From Fig 11, it can be seen that the frequencies decrease as the length parameter increases. It decreases fast in the range 0.5 < *ω*_*m*_ < 0.75. Also, the frequencies of five layered shell are higher than the frequencies of three layered shell.

**Fig 12:**
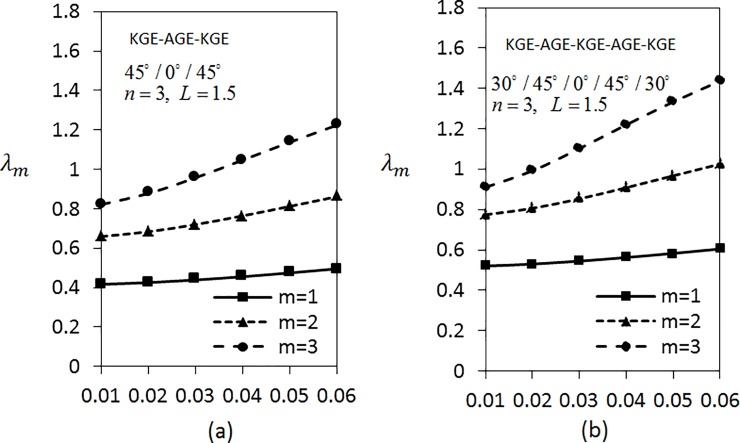
Effect of thickness parameter on frequency for three and five layered symmetric angle-ply shells under C-C boundary conditions.

Next, the frequencies with respect to thickness parameter are analysed as shown in Fig 12. The frequencies of five layered shell in Fig 12(B) are higher than the frequencies of three layered shell in Fig 12(A).

## Conclusion

The vibrational behaviour of layered cylindrical shell with symmetric angle-ply is investigated using spline method. The shell contained a quiescent fluid. The equations of motion of the shell are based on first order shear deformation theory. Investigations on empty and fluid-filled shells show that the frequency value reduces as the fluid term is included. This is due to the fluid in the shell that provides added mass to the shell.

Results show that by increasing the length of the shell, the frequency decreases. In contrast, the frequency increases as the shell thickness increases. Meanwhile, frequency of C-C boundary conditions is higher than the frequency of S-S boundary conditions. It can be concluded that the geometric parameters, material properties, angle orientations, number of layers and boundary conditions significantly affects the frequency of the shell.
